# Nitrogen deposition experiment mimicked with NH_4_NO_3_ overestimates the effect on soil microbial community composition and functional potential in the Eurasian steppe

**DOI:** 10.1186/s40793-022-00441-1

**Published:** 2022-09-12

**Authors:** Tingting Li, Zijia Zhang, Yiping Ma, Yuqian Song, Guojiao Yang, Xingguo Han, Ximei Zhang

**Affiliations:** 1 Key Laboratory of Dryland Agriculture, Ministry of Agriculture, Institute of Environment and Sustainable Development in Agriculture, Chinese Academy of Agricultural Sciences, Beijing, 100081 China; 2 CAS Key Laboratory of Forest Ecology and Management, Erguna Forest-Steppe Ecotone Research Station, Institute of Applied Ecology, Chinese Academy of Sciences, Shenyang, 110016 China; 3 State Key Laboratory of Vegetation and Environmental Change, Institute of Botany, Chinese Academy of Sciences, Beijing, 100093 China; 4 Tianjin Natural History Museum, Tianjin, 300201, China

**Keywords:** Decomposition, Fertilization, Metagenomics, Microbial diversity, Nitrogen deposition

## Abstract

**Background:**

The nitrogenous compound deposited from the atmosphere to the soil is complex, but most field experiments mimic nitrogen deposition with the acid NH_4_NO_3_ alone. Thus, whether the acid and non-acid nitrogenous compounds have similar effects on biodiversity and ecosystem functions remains understudied. We mimicked nitrogen deposition with acidic NH_4_NO_3_ and (NH_4_)_2_SO_4_, and non-acidic urea, slow-released urea and NH_4_HCO_3_ in a temperate steppe, and quantified soil microbial taxonomic and functional gene composition with amplicon sequencing and shotgun metagenomics, respectively.

**Results:**

While NH_4_NO_3_ and (NH_4_)_2_SO_4_ significantly altered the soil microbial taxonomic and functional composition as well as their carbon decomposition potential, the other three compounds had smaller effects.

**Conclusion:**

Our results suggested that previous nitrogen deposition experiments mimicked with NH_4_NO_3_ or (NH_4_)_2_SO_4_ alone may have overestimated the effect on biodiversity and ecosystem functions in the Eurasian steppe and similar ecosystems affected by mainly nonacidic nitrogen deposition.

**Supplementary Information:**

The online version contains supplementary material available at 10.1186/s40793-022-00441-1.

## Background

Due to the agricultural fertilization and combustion of fossil fuels, the nitrogen (N) deposition rate has increased from the pre-industrial levels of approximately 0.1–0.3 to as high as 10 g N/m^2^/year in some developed countries [1–4], and it is predicted that N deposition rate will increase similarly over the next 50 years in many developing countries [3]. N deposition causes a series of ecological consequences, such as changing biological diversity and altering their ecosystem functions. The majority of previous studies focus on the effect of N deposition on plant communities [5–7]. For example, N deposition was found to significantly reduce plant diversity of a mature grassland ecosystem, but had a smaller impact on a nearby degraded ecosystem of the same type [8]. Although soil microbial communities harbor perhaps the highest biodiversity on the planet and they are responsible for many important ecosystem functions such as carbon (C) and nitrogen (N) cycling [9, 10], the effect of N deposition on soil microbial diversity and ecosystem functions remains relatively understudied when compared to the plant communities.

Following the rapid development of amplicon sequencing technologies in the last decade [11, 12], multiple studies have focused on the effects of N deposition on soil microbial taxonomic diversity in various ecosystems and the underlying mechanisms [13, 14]. N deposition was found to shift soil microbial diversity and community composition through elevating soil nutrient content, changing soil pH, as well as altering plant productivity and community structure [15–17]. In addition, N deposition was found to affect soil microbial diversity and community composition through mediating both the deterministic (e.g., environmental filtering and interspecific competition) and stochastic processes (e.g., ecological drift and species migration/colonization) [18–20]. In fact, the functional gene content of soil microbial communities is likely to be more closely associated with ecosystem functioning than the taxonomic diversity [21], and the molecular and computational methods allowing for the quantification of functional gene diversity have recently become widely accessible [10, 22]. However, the effect of N deposition on the functional gene diversity and composition of soil microbial communities still remains relatively underexplored.

Numerous field simulation experiments have been conducted to explore the ecological effects and to reveal the underlying mechanisms of N deposition on biodiversity and ecosystem functions [13, 14]. However, the majority of these experiments (> 50%) mimicked N deposition by adding NH_4_NO_3_ [14, 23, 24]. The component of N deposited from the atmosphere to the soil is complex, and includes compounds with different acid levels. For example, (NH_4_)_2_SO_4_, which is a strong acid and weak base salt, is often the main deposited component in areas with intensive animal husbandry [25], while organic N compounds, which are generally neutral, are the main component in remote ecosystems without anthropogenic influences [26]. Across China, neutral N compounds (e.g., organic N compounds) account for an average of 28% of the total N deposited from the atmosphere, and even up to 50% in the steppe ecosystem in Inner Mongolia [27]. Therefore, determining whether NH_4_NO_3_ and other N compounds have similar effects on soil microbial diversity and ecosystem functions is crucial.

## Methods

### Site description and experimental design

We conducted a three-year field experiment in a steppe ecosystem (50° 10′ 46.1″ N, 119° 22′ 56.4″ E) in Inner Mongolia, China, which is floristically and ecologically representative of much of the Eurasian steppe region. The mean annual precipitation of the site is approximately 363 mm, and the mean annual air temperature is − 2.45 °C [28]. The vegetation is dominated by *Stipa baicalensis, Leymus chinensis and Carex duriuscula*, and the soil is classified as chernozem according to the Food and Agricultural Organization of the United Nations classification [29]. The experiment was established in May 2014, following a randomized complete block design that consisted of six treatments (control, NH_4_NO_3_, (NH_4_)_2_SO_4_, urea, slow-released urea and NH_4_HCO_3_) and four replicates for each treatment. The ambient N deposition rate in this region is < 1.5 g N m^−2^ yr^−1^ [30] and it is predicted to increase in the future [31]. The addition rate for each N compound is 10 g m^−2^ yr^−1^, mimicking the long-term accumulative effects of N deposition in this region. In each plot, N compound was blended with fine sand (500 g) and applied when the grassland turned green (often early June; once every year). Each plot was 10 × 10 m in area.

### Measurement of plant and soil physicochemical indices

In mid-August of 2016 (the time with the highest plant biomass), all aboveground plants were harvested in a 1 × 1 m quadrats from each plot. The plants were sorted by species, and then oven-dried at 65 °C for 48 h and weighted. The total weight of all these plants was calculated as the aboveground plant biomass, and the total species number was counted to represent plant richness. To quantify belowground plant biomass, one soil core with a diameter of 8 cm was collected from each quadrat at 10 cm depth. Roots were collected carefully by a 2 mm sieve, and then oven-dried at 65 °C for 48 h to obtain belowground biomass.

In mid-August of 2016, nine soil cores (10 cm deep, 3.5 cm diameter) were also collected randomly from each plot and mixed to yield one composite sample. Soil samples were stored in a cool box at 4 °C to transport to the laboratory, where the roots and stones were removed using a 2 mm sieve. Part of the composited soil samples was frozen (− 20 °C) for DNA extraction, whereas the remaining portion was used to measure soil pH, soil total organic carbon (TOC) content, total N (TN) content, total phosphorus content, dissolved organic carbon content, NH_4_^+^-N and NO_3_^−^-N content, available phosphorus content and soil moisture. Soil pH was measured in 1:2.5 (W/V) suspensions of soil in deionized water. TOC and TN content were determined by the potassium dichromate-vitriol oxidization method and the Kjeldahl acid-digestion method, respectively [32]. NH_4_^+^-N and NO_3_^−^-N concentrations were determined on a FIAstar 5000 analyzer (Foss Tecator, Denmark) following 2 M KCL (1:50 w/v) extraction for 30 min [32]. Total phosphorus and available phosphorus concentrations were measured by the ammonium molybdate method after persulfate oxidation. The dissolved organic carbon concentration was determined in soil extracts (1:5 soil water ratio) filtered through a 0.45 mm membrane filter using a TOC analyzer (multi NC 2100S, Analytik Jena AG, Jena, Germany). Soil moisture was determined as the weight loss after drying for 24 h at 105 °C.

### Amplicon sequencing and sequence processing

Soil DNA was extracted from 0.25 g fresh soil using the MoBio Power Lyzer Power Soil DNA isolation kit according to the manufacturer’s instructions. To obtain sufficient DNA for subsequent analysis and to overcome the experimental constraints of soil habitat heterogeneity, 4 or 5 extraction replications were mixed to form a composite genetic pool representing the total DNA composition of each sample. The DNA concentration and purity were determined with a NanoDrop 2000 UV–vis spectrophotometer (Thermo Scientific, Wilmington, USA). The quality was checked on 1% agarose gel. Isolated total DNA was stored at − 80 °C for further analysis.

Illumina Miseq sequencing was adopted to quantify bacterial OTU (operational taxonomic unit) diversity. We amplified the fragments of bacterial 16S rRNA V4-V5 using the primers 515F/907R. The primers contained a unique paired barcode sequence for each sample to distinguish samples sequenced in a run. All amplifications were performed in 20 μl reactions containing 4 μl FastPfu Buffer (5 × ; Transgen), 2 μl of 2.5 mM dNTPs, 10 ng of DNA template, 0.4 μl of each primer (5 μM), 0.2 μl of BSA and 0.4 μl of FastPfu Polymerase (Transgen). The PCR conditions are described as follows: 95 ℃ for 3 min (denature), 27 cycles of 94 °C at 30 s, 55 °C for 30 s and 72 °C for 45 s, and a final extension at 72 °C for 10 min. PCR reactions were performed in triplicate. The PCR products were purified by a AxyPrep DNA Gel Extraction Kit (Axygen Biosciences, Union City, CA, USA). Following the quantification by Quantus™ Fluorometer (Promega, USA), the PCR products were pooled at equimolar concentrations for pair-end sequencing with Illumina MiSeq PE300 (Illumina, San Diego, USA) at Majorbio Bio-Pharm Technology Co. Ltd. (Shanghai, China). The raw reads were deposited into NCBI under the project accession number SRP338702.

Raw reads of the 16S rRNA gene were assigned into different sample libraries based on the barcodes and the primers of each read were trimmed. Low quality (Q score < 20) and short reads (length < 300) were discarded. Paired-end reads were merged by FLASH (version 1.2.11) [33] according to at least a 20 bp overlap and < 5% mismatches. Any joined sequences with an ambiguous base or a length of < 300 bp were removed. Thereafter, the OTUs were clustered by UPARSE [34] at a 97% identity [34, 35]. Chimeric sequences were identified and removed using UCHIME [36]. The OTU taxonomic classification of the 16S rRNA gene sequences was assigned using the SILVA database and Ribosomal Database Project Classifier (version 2.11) with 70% confidence estimates [37]. To minimize the effect of unequal sampling on the following calculated indices, 21,093 reads were randomly selected from each sample.

### Shotgun metagenomic sequencing and sequence annotation

Shotgun sequencing was performed on the Illumina Hiseq 2000 platform at Majorbio Bio-pharm Technology Co., Ltd. (Shanghai, China). A sequencing library was prepared by NEXTFLEXRapid DNA-Seq (Bioo Scientific, Austin, TX, USA) following the standard procedure of the manufacturer. Briefly, DNA extract was randomly fragmented to roughly 210 bp using a Covaris M220 instrument (Gene Company Limited, China). Adapters containing the full complement of sequencing primer hybridization sites were ligated to the blunt-end of the fragments. The prepared libraries were then sequenced. The sequence data have been deposited in the NCBI Short Read Archive database (Accession Number: SRP338302). Shotgun sequencing resulted in 89.8 ± 2.6 million sequences (mean ± one standard error) or 13.5 ± 0.4 Giga base pairs (Gbp) of sequences per sample.

Paired-end shotgun metagenomic sequences were trimmed and quality controlled using Sickle (https://github.com/najoshi/sickle) and SeqPrep (https://github.com/jstjohn/SeqPrep) with default parameters [22]. Clean reads were then merged using FLASH [33]. Following this, reads > 50 bp were retained and aligned to eggNOG 5.0 and Swiss-Prot [38] using Blastx (E-value < 10^–5^, coverage > 80%, identity > 30%). A count matrix of the summarized COG (cluster of orthologous groups of proteins) function terms was obtained to evaluate the functional richness and composition of the soil microbial community. Another count matrix was generated to summarize the occurrence of each Swiss-Prot entry in each sample. The corresponding Gene Ontology Annotations of functions and processes for each Swiss-Prot entry were obtained from the uniprot_sprot.dat file provided on http://www.uniprot.org/downloads (downloaded on July 2017). To estimate the effects of N compound on soil carbon degradation potential of soil microbial communities, we focused on the genes involved in the soil carbon decomposition.

### Statistical analysis

The differences in soil physicochemical indices, microbial diversity indices and the relative abundance of carbon decomposition genes were determined by a general linear model, followed by the least significant difference (*P* < 0.05) test using SPSS (version 21.0). Bray–Curtis distances calculated from the relative abundances of OTUs and COG terms were used to assess the pairwise distance between taxonomic and functional community composition, respectively. Bray–Curtis distances calculated from the biomass of plant species were used to represent the dissimilarity in plant community composition. Principle co-ordinates analysis (PCoA) was used to visualize changes in microbial taxonomic and functional community composition. PERMANOVA (permutational multivariate analysis of variance) was performed to test significant changes in microbial taxonomic and functional community composition. The Mantel test was used to reveal the relationship between the soil microbial community structure and environmental factors (soil physicochemical and plant community indices). The spearman correlation was used to analyze the effects of environmental variables on compositional PCoA axis 1 and the relative abundance of dominant bacterial taxa. The statistical significance was determined at *P* < 0.05 for all analyses. Partial least squares path modeling (PLS-PM) was performed to further evaluate the direct and indirect effects of environmental factors (soil properties and the plant biomass) on soil microbial taxonomic and functional communities (R plspm package). The path coefficients and explained variability (R^2^) reflected the cause-and-effect relationships among observed and latent variables. A goodness of fit (GOF) test was used to evaluate the predictive power of the model [39]. Various statistical analyses were carried out using R v4.1.0 software unless otherwise indicated.

## Results and discussion

General linear model revealed that among these measured indices, the treatments only significantly affected above- and below-ground plant biomass, soil pH, NH_4_^+^-N and NO_3_^−^-N content (Table 1). Multiple comparisons further revealed that the five N compounds had significantly different effects on these indices (Table 1). In particular, when compared to the control, only NH_4_NO_3_, (NH_4_)_2_SO_4_ and NH_4_HCO_3_ significantly decreased belowground biomass, while only NH_4_NO_3_, (NH_4_)_2_SO_4_ and urea significantly decreased soil pH. NH_4_NO_3_ and (NH_4_)_2_SO_4_ are strong acid and weak base salts, NH_4_HCO_3_ is a weak acid and weak base salt, and urea and slow-released urea are organic N compounds. Thus, NH_4_NO_3_ and (NH_4_)_2_SO_4_ had the largest acidification effect on the soil, inhibiting ammonia volatilization and plant root growth, resulting in higher NH_4_^+^-N content and lower belowground plant biomass.

**Table 1:** Effects of different forms of N addition on soil, plant and microbial properties

Indices	Mean (± se) under each treatment						General lineal models	
	Control	NH_4_NO_3_	Slow-released urea	Urea	NH_4_HCO_3_	(NH_4_)_2_SO_4_	Block	N addition
Soil total organic carbon content (g kg^−1^)	42.509 (4.332)	35.292 (1.609)	40.679 (2.912)	40.329 (2.287)	37.47 (1.409)	37.462 (0.622)	0.304	0.358
Soil total N content (g kg^−1^)	2.555 (0.070)	2.593 (0.137)	2.940 (0.169)	2.753 (0.072)	2.705 (0.038)	2.788 (0.104)	0.325	0.183
C/N	16.608 (1.578)	13.72 (0.895)	13.905 (0.923)	14.643 (0.697)	13.88 (0.702)	13.512 (0.677)	0.645	0.302
Soil total phosphorus content (g kg^−1^)	0.118 (0.008)	0.108 (0.001)	0.111 (0.003)	0.108 (0.003)	0.105 (0.002)	0.109 (0.002)	0.421	0.383
Soil NO_3_^−^-N content (mg kg^−1^)	11.295 (1.556)^c^	29.540 (5.842)^b^	49.817 (2.790)^a^	45.975 (5.173)^ab^	29.924 (1.236)^b^	34.050 (5.987)^ab^	0.43	< 0.001
Soil NH_4_^+^-N content (mg kg^−1^)	1.673 (0.316)^d^	28.950 (6.953)^b^	22.750 (3.562)^bc^	6.513 (1.379)^cd^	2.213 (0.224)^d^	115.240 (12.247)^a^	0.237	< 0.001
Soil dissolved organic carbon content (mg g^−1^)	351.345 (61.620)	258.636 (50.380)	260.702 (39.512)	235.843 (34.609)	216.965 (28.755)	222.135 (16.549)	0.414	0.268
Soil available phosphorus content (mg kg^−1^)	6.450 (1.393)	6.775 (0.642)	7.050 (0.972)	6.650 (0.096)	6.900 (0.670)	6.275 (0.256)	0.486	0.984
Soil pH	6.888 (0.081)^a^	6.590 (0.079)^b^	6.735 (0.093)^ab^	6.548 (0.094)^b^	6.668 (0.029)^ab^	6.030 (0.094)^c^	0.111	< 0.001
Soil moisture (%)	15.392 (0.271)	15.414 (0.264)	15.705 (0.229)	14.791 (0.747)	15.401 (0.179)	15.799 (0.401)	0.791	0.633
Aboveground plant biomass (g m^−2^)	130.283 (11.184)^b^	240.610 (40.884)^a^	209.565 (25.066)^ab^	214.028 (27.775)^ab^	169.780 (19.101)^ab^	146.265 (23.029)^b^	0.178	0.040
Belowground plant biomass (g m^−2^)	110.650 (6.916)^a^	57.340 (5.901)^b^	115.420 (27.360)^a^	93.663 (6.534)^ab^	63.250 (6.680)^b^	48.168 (10.128)^b^	0.598	0.009
Plant species richness	14.750 (2.462)	16.500 (1.500)	13.000 (1.291)	11.250 (1.031)	13.750 (1.652)	15.750 (3.227)	0.638	0.408
Microbial biomass carbon (mg g^−1^)	0.927 (0.081)	0.779 (0.067)	0.957 (0.091)	0.873 (0.069)	0.993 (0.037)	0.810 (0.044)	0.988	0.313

Different letters within a row indicate significant differences between treatments (*P* < 0.05)

**Fig. 1:**
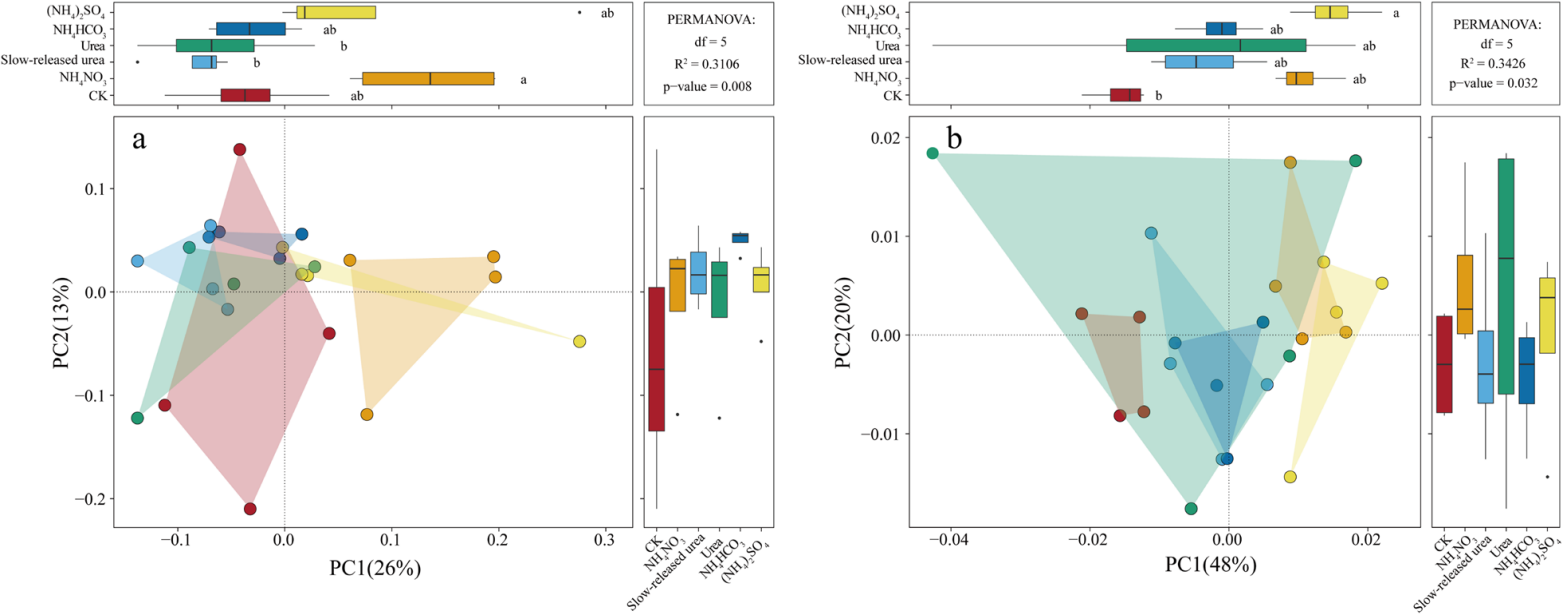
Principal coordinate analysis (PCoA) of soil bacterial composition **a** and soil microbial functional gene composition **b** under different N forms. PERMANOVA: permutational multivariate analysis of variance

**Fig. 2:**
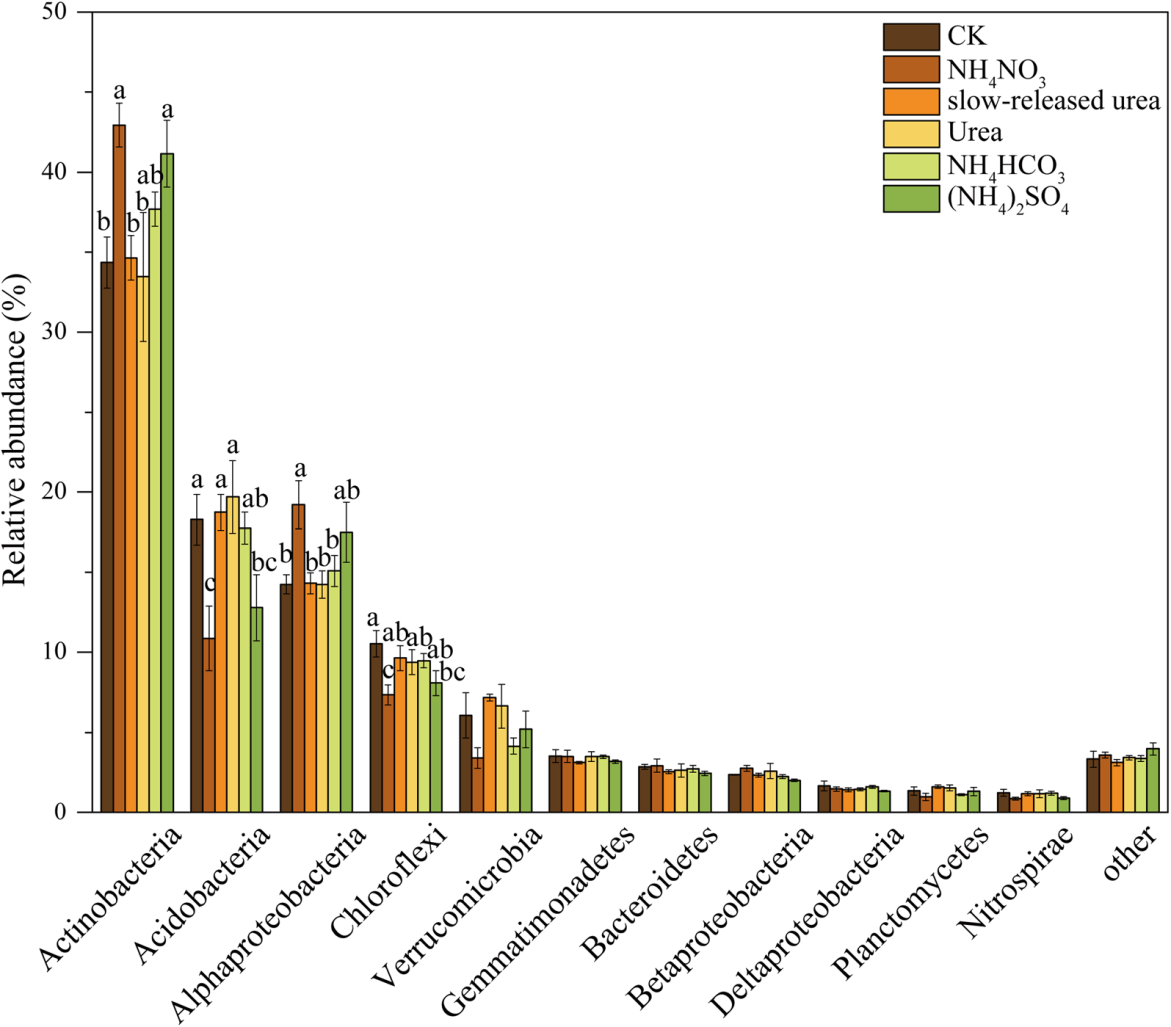
Relative abundances of dominant bacterial phyla (> 1%) under different forms of N addition. Results are reported as mean ± se (n = 4). Different letters indicate significant (*P* < 0.05) differences among treatments

Permutational multivariate analysis of variance (PERMANOVA) revealed that the treatments had a non-significant effect on plant community composition based on species relative biomass (*P* > 0.05). In contrast, they had a significant effect on soil bacterial taxonomic composition (*P* < 0.05; Fig. 1a). In particular, multiple comparisons revealed that NH_4_NO_3_ and (NH_4_)_2_SO_4_ were statistically different from the others (Additional file 1: Table S1), which was also reflected in the axis 1 of the principal coordinate analysis (PCoA; Fig. 1a). More specifically, NH_4_NO_3_ and (NH_4_)_2_SO_4_ significantly increased the relative abundances of phylum *Actinobacteria* and *Alphaproteobacteria*, while decreased those of *Acidobacteria* and *Chloroflexi* (Fig. 2), agreeing with other studies [10, 16]. The mantel test and correlation analysis revealed that soil NH_4_^+^-N content was the main factor controlling soil bacterial community composition and the relative abundances of the main bacterial taxa (Table 2, Additional file 2: Table S2).

**Table 2:** Relationship between soil microbial community composition (or C-decomposition genes composition) and environmental attributes revealed by Mantel test

	Bacterial community composition		Microbial functional composition		C-decomposition genes composition	
	R	*P*	r	*P*	r	*P*
Soil total N content	− 0.118	0.837	− 0.048	0.663	0.079	0.218
Soil total organic carbon content	0.146	0.123	0.159	0.102	0.168	0.086
Soil NH_4_^+^-N content	**0.138**	0.048	**0.201**	0.003	**0.222**	0.005
Soil NO_3_^−^-N content	0.201	0.052	0.073	0.201	0.156	0.064
Soil pH	0.075	0.274	**0.253**	0.028	**0.292**	0.010
Aboveground plant biomass	− 0.058	0.743	− 0.058	0.755	− 0.053	0.742
Belowground plant biomass	0.131	0.122	**0.240**	0.010	**0.255**	0.008

Bold r values indicate significant correlation

**Fig. 3:**
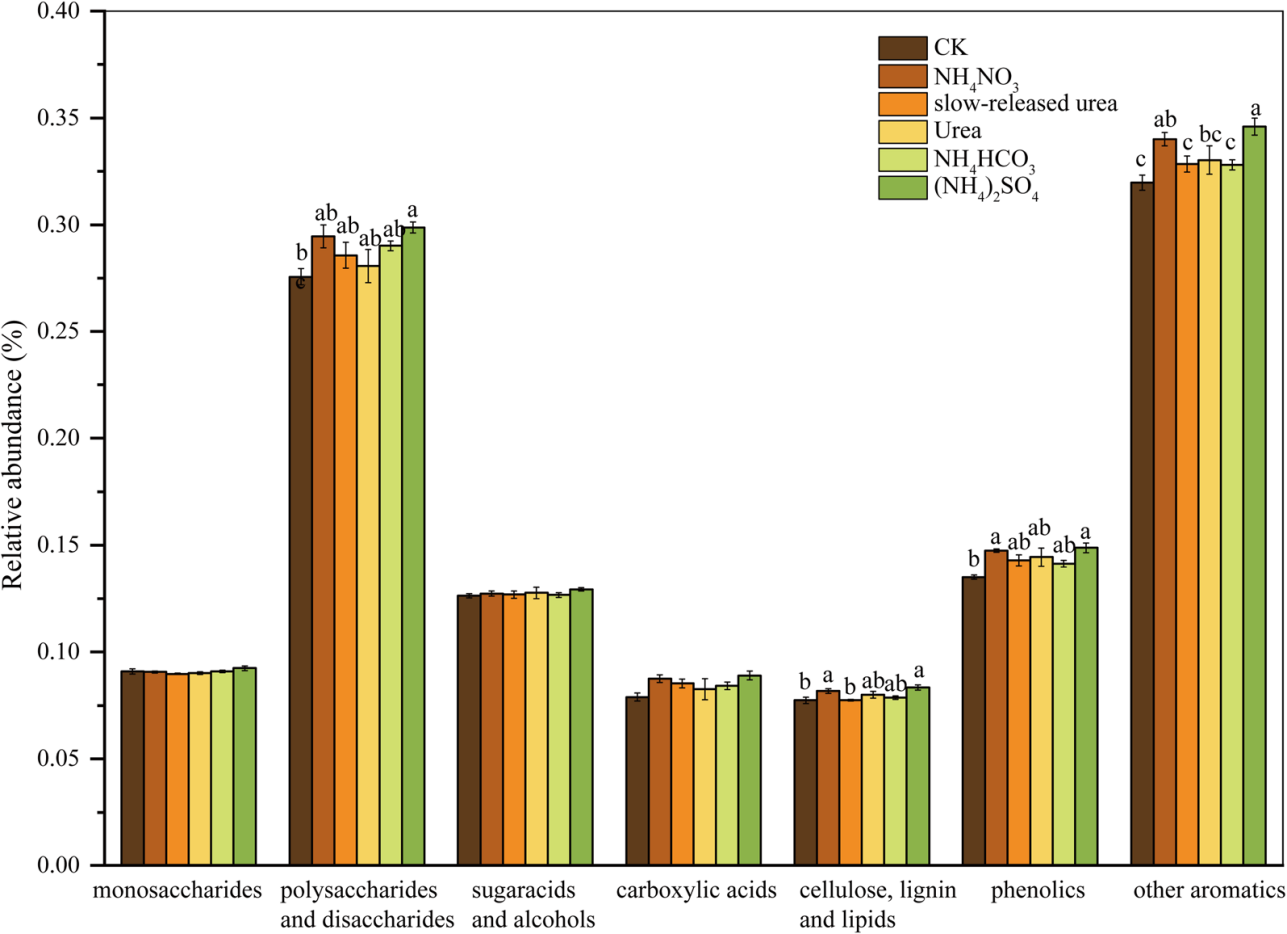
Relative abundances of functional genes involved in C degradation. The complexity of carbon is presented in order from labile to recalcitrant. Results are reported as the mean ± se (n = 4). Different letters indicate significant (*P* < 0.05) differences among treatments

Similar to the taxonomic composition, PERMANOVA revealed that the treatments had a significant effect on soil microbial functional gene composition (*P* < 0.05; Fig. 1b), and multiple comparisons revealed that NH_4_NO_3_ and (NH_4_)_2_SO_4_ were statistically different from the other compounds (Additional file 1: Table S1), which was also reflected in the PCoA axis 1 (Fig. 1b). Moreover, NH_4_NO_3_ and (NH_4_)_2_SO_4_ significantly increased the relative abundances of genes for the degradation of relatively recalcitrant organic matters, including aromatics, phenolics, cellulose, lignin, lipids, and polysaccharides (Fig. 3). The mantel test revealed that functional gene composition and C-decomposition genes were both correlated with soil NH_4_^+^-N content, soil pH and belowground biomass (Table 2).

Our results demonstrated that acid N compounds (NH_4_NO_3_ and (NH_4_)_2_SO_4_) had stronger effects on soil microbial community and functional potential than the non-acid N compounds (urea, slow-released urea and NH_4_HCO_3_). Consistent with our study, Wang et al. [40] in 2018 revealed that NH_4_NO_3_ reduced the soil microbial bacterial/fungi ratio, while urea had no significant effect on the ratio in a temperate forest in China. Previous meta-analyses also demonstrated the NH_4_NO_3_ exerted a negative impact on microbial biomass and bacterial diversity at the global scale, while urea had no significant impact [24, 41]. Similarly, Wang et al. [42] in 2019 reported that the abundances of the majority of N cycling and C decomposition genes under (NH_4_)_2_SO_4_ addition were significantly higher than those under neutral N compound (KNO_3_) addition, which was due to the lower soil pH and higher soil ammonium content caused by (NH_4_)_2_SO_4_ addition.

As previously mentioned, NH_4_NO_3_ and (NH_4_)_2_SO_4_ are strong acid and weak base salts, thus leading to a stronger soil acidification effect after application [42, 43]. The acidification can impact soil bacterial communities directly through influencing bacterial growth. The maximum values of bacterial growth were observed to be in neutral soils [44, 45]. Therefore, bacterial growth would be inhibited in acidic soils, resulting in the exclusion of some intolerant bacterial groups [46–48] and a shift in the bacterial function. Alternatively, the higher acidification effects of acid N compounds may impact the bacterial community indirectly through inhibiting ammonia volatilization, nitrification, and the growth of plant roots [49–51] (Additional file 3: Fig. S1). Ammonium is preferentially used by soil organisms due to its low energy cost [52], and has thus been identified as the determinant factor for soil bacterial community composition [53]. In addition, the carbon originating from rhizodeposition is pivotal for soil microbial growth [54–56], while its reduction would definitely impact bacterial growth and their carbon metabolism. Interestingly, we indeed detected that NH_4_NO_3_ and (NH_4_)_2_SO_4_ had stronger effects on the bacterial potential function relating to the carbon metabolism. Taken together, NH_4_NO_3_ and (NH_4_)_2_SO_4_ had the largest soil acidification effect and higher soil NH_4_^+^-N content and also decreased plant root biomass, which together led to the changes in soil microbial taxonomic composition, functional gene composition, and also the degradation potential of soil organic matter.

Overall, our results demonstrated that acid N compounds (NH_4_NO_3_ and (NH_4_)_2_SO_4_) had stronger effects on soil microbial community and functional potential than the non-acid N compounds (urea, slow-released urea and NH_4_HCO_3_). Thus, it would be unsuitable to mimic the effects of N deposition by adding NH_4_NO_3_ or (NH_4_)_2_SO_4_ alone in areas where non-acidic N compounds (e.g., organic N compounds) contribute the most of N deposition, such as the Eurasian steppe [27]. In other words, the N deposition experiments simulated with NH_4_NO_3_ alone are likely to have overestimated the effect on soil microbial diversity and functions in the Eurasian steppe. For example, when the addition of NH_4_HCO_3_ increased the relative abundances of phylum *Actinobacteria* by only 9.68% (Table 3), adding NH_4_NO_3_ resulted in an increase of 24.97%. Hence if the NH_4_NO_3_ addition is used to mimic N deposition in areas where the actual deposited N is NH_4_HCO_3_, the effect on the *Actinobacteria* relative abundance will be overestimated by 157% ((24.97–9.68)/9.68%). Similarly, when the NH_4_HCO_3_ addition increased the relative abundances of cellulose-degradation genes by only 1.70% (Table 3), adding NH_4_NO_3_ resulted in an increase of 5.63%, and consequently, a 231% ((5.63–1.70)/1.70%) overestimation.

**Table 3:** Percent changes in the relative abundances of bacterial phyla and C-decomposition gene categories under different N compound addition treatments when compared with the control

Bacterial phyla/gene categories	NH_4_NO_3_ (%)	Slow-released urea (%)	Urea (%)	NH_4_HCO_3_ (%)	(NH_4_)_2_SO_4_ (%)
Actinobacteria	24.97	0.83	− 2.63	9.68	19.79
Acidobacteria	− 40.48	2.54	7.83	− 2.88	− 29.95
Alphaproteobacteria	34.66	0.52	− 0.02	5.87	22.81
Chloroflexi	− 30.27	− 8.54	− 11.01	− 10.09	− 23.36
Verrucomicrobia	− 43.85	17.99	9.26	− 31.72	− 14.36
Gemmatimonadetes	− 0.44	− 11.23	− 0.64	− 0.81	− 9.68
Bacteroidetes	2.54	− 11.12	− 7.62	− 4.54	− 14.37
Betaproteobacteria	17.38	− 1.01	10.11	− 4.70	− 14.60
Deltaproteobacteria	− 11.89	− 14.54	− 12.68	− 4.16	− 19.84
Planctomycetes	− 27.42	19.52	15.53	− 18.46	− 2.13
Nitrospirae	− 30.23	− 5.47	− 5.18	− 3.36	− 29.17
Other	7.81	− 6.35	3.07	1.39	19.58
Monosaccharides	− 0.22	− 1.25	− 0.82	0.01	1.66
Polysaccharides and disaccharides	6.87	3.65	1.83	5.27	8.36
Sugar acids and alcohols	0.71	0.36	0.99	0.21	2.24
Carboxylic acids	10.83	8.02	4.63	6.76	12.73
Cellulose,lignin and lipids	5.63	0.20	3.45	1.70	7.76
Phenolics	9.24	5.87	7.00	4.66	10.16
Other aromatics	6.33	2.70	3.28	2.57	8.21

## Conclusion

In this study, we investigated whether NH_4_NO_3_ and other N compounds had similar effects on microbial communities and the corresponding functions. Acidic NH_4_NO_3_ and (NH_4_)_2_SO_4_ significantly altered soil microbial taxonomic and functional composition as well as their carbon decomposition potential, while non-acidic urea, slow-released urea and NH_4_HCO_3_ had smaller effects. This indicates that previous N deposition experiments mimicked with acidic NH_4_NO_3_ alone in the Eurasian steppe and similar ecosystems may have overestimated the effect on biodiversity and ecosystem functions, and that the actual deposited N compound or even the mixtures of different N compounds should be used to simulate atmospheric N deposition in future studies. Our study was conducted in a meadow steppe with simulated N deposition for just three years. Therefore, the results in this study may differ from those of long-term experiments and other ecosystems. In particular, the effect of these non-acidic N compounds on soil microbial diversity and ecosystem functions may turn to be more significant as the treatment time lasts much longer. Further work is required to test the generality of these results in long-term experiments and other ecosystems.

## Supplementary Information

**Additional file 1: Table S1.** Pairwise PERMANOVA of microbial taxonomic (upper right) and functional (lower left) community composition under different treatments. PERMANOVA: permutational multivariate analysis of variance. Data are the p values.

**Additional file 2: Table S2.** Correlation between relative abundance of dominant bacterial taxa and environmental variables.

**Additional file 3: Fig. S1.** Partial least squares path models (PLS-PM) for bacterial communities and C-decomposition potential.

## Data Availability

The amplicon sequencing and shotgun metagenomic sequencing data in this study was deposited in NCBI under the project accession number SRP338702 and SRP338302, respectively.

## Abbreviations

PERMANOVA: Permutational multivariate analysis of variance
PCoA: Principal coordinate analysis
C-decomposition: Carbon decomposition
TOC: Total organic carbon
TN: Total nitrogen
