# Supplementary material for: Nitrogen deposition experiment mimicked with NH4NO3 overestimates the effect on soil microbial community composition and functional potential in the Eurasian steppe
Source: Environ Microbiome. 2022 Sep 12;17:49. doi: 10.1186/s40793-022-00441-1 (PMC9469546; doi:10.1186/s40793-022-00441-1)
Supplement: Supplementary file 1 — Additional file 1: Table S1. Pairwise PERMANOVA of microbial taxonomic (upper right) and functional (lower left) community composition under different treatments. PERMANOVA: permutational multivariate analysis of variance. Data are the p values. [file 40793_2022_441_MOESM1_ESM.docx]

**Table S1.** Pairwise PERMANOVA of microbial taxonomic (upper right) and functional (lower left) community composition under different treatments. PERMANOVA: permutational multivariate analysis of variance. Data are the *p* values.

|  | CK | NH₄NO₃ | Slow-released urea | Urea | NH₄HCO₃ | (NH₄)₂SO_₄_ |
| --- | --- | --- | --- | --- | --- | --- |
| CK |  | 0.03 | 0.38 | 0.69 | 0.22 | 0.11 |
| NH₄NO₃ | 0.02 |  | 0.03 | 0.09 | 0.05 | 0.74 |
| Slow-released urea | 0.21 | 0.03 |  | 0.93 | 0.03 | 0.05 |
| Urea | 0.74 | 0.33 | 0.99 |  | 0.34 | 0.06 |
| NH₄HCO₃ | 0.04 | 0.05 | 0.97 | 0.94 |  | 0.03 |
| (NH₄)₂SO_₄_ | 0.04 | 0.68 | 0.05 | 0.14 | 0.05 |  |
