# Supplementary material for: Nitrogen deposition experiment mimicked with NH4NO3 overestimates the effect on soil microbial community composition and functional potential in the Eurasian steppe
Source: Environ Microbiome. 2022 Sep 12;17:49. doi: 10.1186/s40793-022-00441-1 (PMC9469546; doi:10.1186/s40793-022-00441-1)
Supplement: Supplementary file 2 — Additional file 2: Table S2. Correlation between relative abundance of dominant bacterial taxa and environmental variables. [file 40793_2022_441_MOESM2_ESM.docx]

**Table S2** Correlation between relative abundance of dominant bacterial taxa and environmental variables.

| Bacterial taxa | TN | TOC | NH_4_^+^-N | NO_3_^-^-N | PH | AGB | BGB |
| --- | --- | --- | --- | --- | --- | --- | --- |
| Acidobacteria | 0.087 | 0.084 | **-0.556**** | 0.138 | 0.251 | 0.095 | **0.526**** |
| Actinobacteria | -0.062 | -0.168 | **0.543**** | -0.07 | -0.259 | 0.003 | **-0.595**** |
| Chloroflexi | 0.105 | 0.210 | -0.361 | -0.022 | 0.307 | 0.014 | **0.522**** |
| Alphaproteobacteria | -0.226 | -0.052 | **0.402*** | -0.158 | -0.283 | -0.158 | **-0.54**** |

Note: TN: total nitrogen; TOC: total organic carbon; NH_4_^+^-N: ammonium nitrogen; NO_3_^-^ -N: nitrate nitrogen; AGB: aboveground biomass; BGB: belowground biomass. Bold values indicate significant correlation (***p*<0.01; **p*<0.05)
