# Supplementary figures and images for: Nitrogen deposition experiment mimicked with NH4NO3 overestimates the effect on soil microbial community composition and functional potential in the Eurasian steppe
Source: Environ Microbiome. 2022 Sep 12;17:49. doi: 10.1186/s40793-022-00441-1 (PMC9469546; doi:10.1186/s40793-022-00441-1)

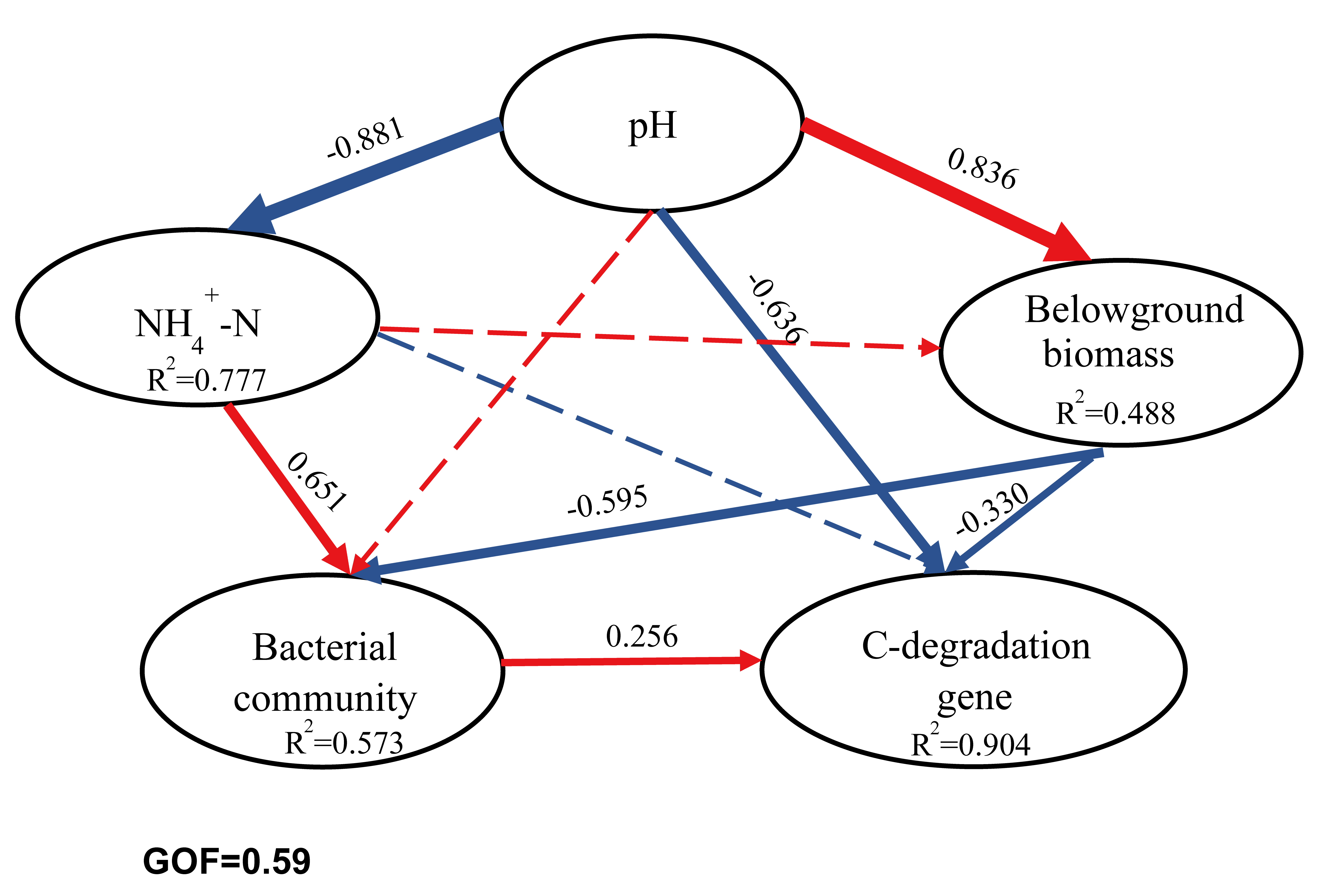

Supplement: Supplementary file 3 — Additional file 3: Fig. S1. Partial least squares path models (PLS-PM) for bacterial communities and C-decomposition potential. [file 40793_2022_441_MOESM3_ESM.tif]
